# RETRACTION: NEAT1 Enhances MPP + ‐Induced Pyroptosis in a Cell Model of Parkinson's Disease via Targeting miR‐5047/YAF2 Signaling

**DOI:** 10.1002/iid3.70208

**Published:** 2025-05-28

## Abstract

**RETRACTION:** H. Shen, H. Song, S. Wang, D. Su and Q. Sun, “NEAT1 Enhances MPP + ‐Induced Pyroptosis in a Cell Model of Parkinson's Disease via Targeting miR‐5047/YAF2 Signaling,” *Immunity, Inflammation and Disease* 11, no. 6 (2023): e817, https://doi.org/10.1002/iid3.817.

The above article, published online on 23 June 2023 in Wiley Online Library (wileyonlinelibrary.com), has been retracted by agreement between the journal Editor‐in‐Chief, Marc Veldhoen; and John Wiley & Sons, Ltd. The retraction has been agreed upon due to the ASC western blot bands in Figure 1 C being found duplicated in a previously published article, representing a different scientific context. The authors were contacted for comment and supporting data but did not respond. The editors consider the results and conclusions of this article unreliable. The authors were informed of the retraction.

**RETRACTION:** H. Shen, H. Song, S. Wang, D. Su and Q. Sun, “NEAT1 Enhances MPP + ‐Induced Pyroptosis in a Cell Model of Parkinson's Disease via Targeting miR‐5047/YAF2 Signaling,” *Immunity, Inflammation and Disease* 11, no. 6 (2023): e817, https://doi.org/10.1002/iid3.817.

The above article, published online on 23 June 2023 in Wiley Online Library (wileyonlinelibrary.com), has been retracted by agreement between the journal Editor‐in‐Chief, Marc Veldhoen; and John Wiley & Sons, Ltd. The retraction has been agreed upon due to the ASC western blot bands in Figure 1 C being found duplicated in a previously published article, representing a different scientific context. The authors were contacted for comment and supporting data but did not respond. The editors consider the results and conclusions of this article unreliable. The authors were informed of the retraction.

